# The Role of Long Non-Coding RNAs in Endometriosis

**DOI:** 10.3390/ijms222111425

**Published:** 2021-10-22

**Authors:** Quanah J. Hudson, Katharina Proestling, Alexandra Perricos, Lorenz Kuessel, Heinrich Husslein, René Wenzl, Iveta Yotova

**Affiliations:** Department of Obstetrics and Gynecology, Medical University of Vienna, Waehringer Guertel 18-20, A-1090 Vienna, Austria; quanah.hudson@univie.ac.at (Q.J.H.); katharina.proestling@meduniwien.ac.at (K.P.); alexandra.perricos@meduniwien.ac.at (A.P.); lorenz.kuessel@meduniwien.ac.at (L.K.); heinrich.husslein@meduniwien.ac.at (H.H.); rene.wenzl@meduniwien.ac.at (R.W.)

**Keywords:** endometriosis, long non-coding RNAs, lncRNAs, sponging, ceRNA, chromatin, chronic pain

## Abstract

Endometriosis is a chronic gynecological disorder affecting the quality of life and fertility of many women around the world. Heterogeneous and non-specific symptoms may lead to a delay in diagnosis, with treatment options limited to surgery and hormonal therapy. Hence, there is a need to better understand the pathogenesis of the disease to improve diagnosis and treatment. Long non-coding RNAs (lncRNAs) have been increasingly shown to be involved in gene regulation but remain relatively under investigated in endometriosis. Mutational and transcriptomic studies have implicated lncRNAs in the pathogenesis of endometriosis. Single-nucleotide polymorphisms (SNPs) in lncRNAs or their regulatory regions have been associated with endometriosis. Genome-wide transcriptomic studies have identified lncRNAs that show deregulated expression in endometriosis, some of which have been subjected to further experiments, which support a role in endometriosis. Mechanistic studies indicate that lncRNAs may regulate genes involved in endometriosis by acting as a molecular sponge for miRNAs, by directly targeting regulatory elements via interactions with chromatin or transcription factors or by affecting signaling pathways. Future studies should concentrate on determining the role of uncharacterized lncRNAs revealed by endometriosis transcriptome studies and the relevance of lncRNAs implicated in the disease by in vitro and animal model studies.

## 1. Introduction

Endometriosis is a chronic inflammatory disorder defined by endometrial-like lesions growing ectopically outside of the uterus that affects many reproductive-age women worldwide. The disease has heterogeneous symptoms that may not be specific to the disease, such as different types of chronic gynecological pain and reduced fertility. The cause of the disease remains unclear, but lesions are thought to originate from endometrial cells that escape the uterus [1], although the presence of rare endometriosis-like lesions in males supports a trans-differentiation origin [2]. Endometriosis is thought to be an immunological disease, first because lesions must evade the immune system in order to establish and develop, and second because the inflammatory nature of the disease accounts for many of the symptoms [3]. Additionally, a meta-analysis has found that there is some association between endometriosis and a risk for developing an autoimmune disease [4]. Treatment options for the disease remain limited to laparoscopic surgery to remove lesions and hormone-based treatments that are not compatible with pregnancy [3]. Given that diagnosis is often delayed due to the non-specific symptoms and that there are limited treatment options, there is a need to better understand the molecular pathogenesis of the disease in order to identify key players that may be used as biomarkers for diagnosis or as targets for new treatments. One class of molecules that may play a role in the disease are long non-coding RNAs (lncRNAs).

LncRNAs are a class of genes that since the advent of high-throughput sequencing technology have been revealed to be much more numerous than was previously realized. Currently over 16,000 human lncRNAs are annotated by the GENCODE project, while some other studies have indicated that the total number may be over 100,000 [5]. Although most of these lncRNAs remain uncharacterized, an increasing number have been shown to have a biological function, often involved in gene regulation [6]. LncRNAs remain relatively under investigated in endometriosis, but given their roles in development and disease in other contexts [7], it is reasonable to assume that there may be lncRNAs that also play a role in endometriosis.

**Figure 1:**
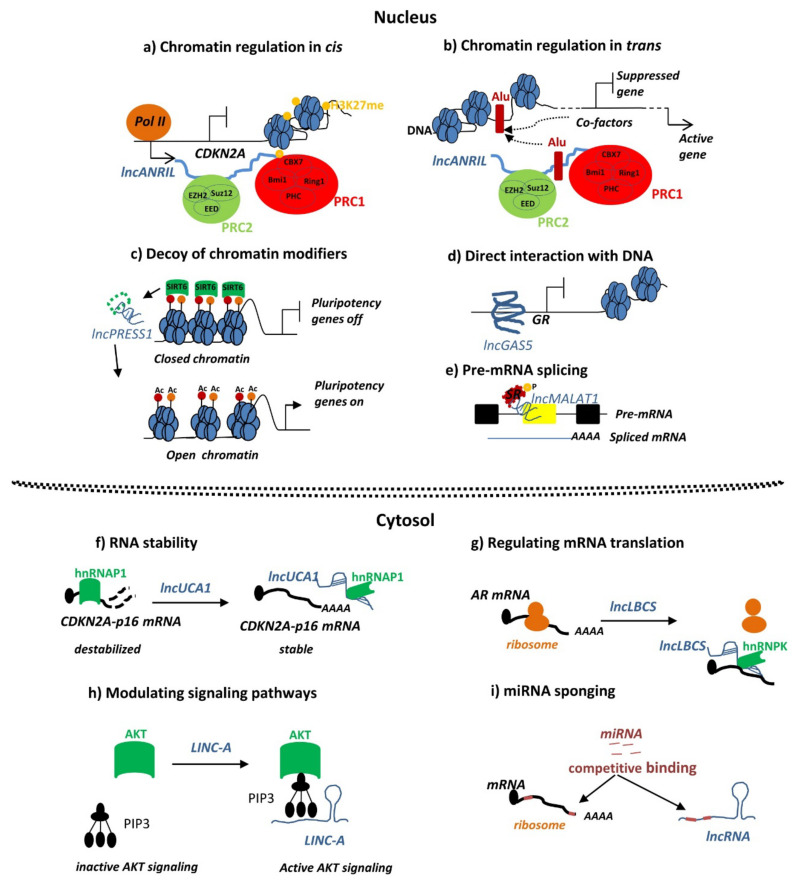
Description of the general mechanisms of the lncRNA actions in the nucleus (**a**–**e**) or in the cytosol (**f**–**i**). (**a**) Regulation in *cis* by lncRNA *ANRIL*, which mediates polycomb repressive complex (PRC) 1 and 2 recruitment to the promoter of the neighboring *CDKN2A* and *CDKN2B* genes, thereby controlling their expression [8]. (**b**) Regulation in *trans* by *ANRIL*, which acts through Alu sequences to recruit PRC1 and PRC2 complexes to distant targets [9]. (**c**) The embryonic stem cell–specific lncRNA *lncPRESS1* sequesters the histone deacetylase Sirtuin 6 (SIRT6) from the promoters of numerous pluripotency genes. *LncPRESS1* keeps histone H3 acetylated, thereby activating the transcription of pluripotency genes. During differentiation or following depletion of *lncPRESS1*, SIRT6 localizes to the chromatin, blocking the transcription of pluripotency genes [10]. (**d**) The lncRNA *GAS5* folds into a DNA-like structure and binds to the glucocorticoid receptor (GR), thereby inhibiting its transcriptional activity [11]. (**e**) The nuclear-retained lncRNA *MALAT1* can regulate alternative splicing by modulating the phosphorylation of the SR splicing factor [12]. (**f**) In the cytosol, the lncRNA *urothelial carcinoma associated 1* (*UCA1*) stabilizes *CDKN2A-p16* mRNA by sequestering the heterogeneous nuclear ribonucleoprotein A1 (hnRNPA1) [13]. (**g**) LncRNA *LBCS* suppresses the androgen receptor (AR) translation efficiency by forming a complex with hnRNPK and *AR* mRNA [14]. (**h**) The *lncRNA for kinase activation* (*LINK-A*) directly interacts with the AKT pleckstrin homology domain and PIP3 facilitating AKT–PIP3 interaction and consequent enzymatic activation [15]. (**i**) The lncRNA *PVT1* sponges *miR-503* to upregulate *ARL2* expression in cervical cancer [17].

LncRNAs may exert their function in either the nucleus or the cytoplasm via a variety of mechanisms. In the nucleus, lncRNAs may act as epigenetic gene regulators by recruiting chromatin remodeling or modification complexes to target gene promoters either in *cis* (Figure 1a) [8] and/or in *trans* to activate or suppress their transcription (Figure 1b) [9]. LncRNAs can act as decoys of specific chromatin modifiers by sequestering them from the promoters of target genes (Figure 1c) [10]. In other contexts, lncRNAs may act as transcriptional regulators by competing with a transcription factor for binding DNA and/or by binding its DNA binding domain (Figure 1d) [11]. LncRNAs have also been associated with the regulation of pre-mRNA alternative splicing in the nucleus, thereby affecting which isoforms predominate (Figure 1e) [12]. In the cytoplasm, lncRNAs are involved in post-transcriptional regulation processes that can affect the stability of transcripts (Figure 1f) [13] or whether a transcript is translated into a protein or not (Figure 1g) [14]. LncRNAs can also be involved in the modulation of cell signaling pathways by binding to signaling proteins and affecting their activation state (Figure 1h) [15]. Finally, a common mechanism whereby lncRNAs are thought to affect mRNA abundance is by acting as so-called “sponges” of miRNAs to reduce miRNA-mediated degradation. Here, lncRNAs may share miRNA target sequences with mRNAs, thereby reducing miRNA availability to target mRNAs (Figure 1i) [16,17].

These studies show that lncRNAs can act at multiple subcellular sites to affect various aspects of cell biology, although in most cases they affect either transcription or processing of mRNAs and their steady state levels in the cell. That is, in a broad sense, they regulate other genes. An increasing body of literature implicates abnormal expression of specific lncRNAs in disease, particularly in different types of cancer [18], but also in a variety of other diseases including neural disorders [19], cardiovascular disease [20], and diabetes [21]. Evidence for the involvement of individual lncRNAs in the pathogenesis of these diseases ranges from a correlation with dysregulated lncRNA expression to detailed mechanistic studies that indicate a functional role in the disease. The implication from these studies and others is that lncRNAs are important players in the pathogenesis of many diseases, suggesting that there may be lncRNAs that play a role in endometriosis. Furthermore, a number of studies in have shown that lncRNAs can either be regulated by estrogen-dependent enhancers [22] or that lncRNAs can regulate estrogen receptor expression [23]. As endometriosis is an estrogen-dependent disease [3], these studies give further impetus to investigate the role of lncRNAs in the disease. Given this evidence for the role of lncRNAs in other contexts, in this review, we aim to provide a comprehensive picture of the current state of knowledge over the role of lncRNAs in endometriosis.

## 2. Aims and Methodology of This Review

In this review, we aim to provide a comprehensive picture of the current knowledge of the role of lncRNAs in the pathogenesis of endometriosis and the mechanisms by which they affect the disease. We included patient studies to detect lncRNAs that have been associated with endometriosis, along with in vitro studies and in vivo animal studies to analyze the mechanism of action and function of lncRNAs in the disease. To gather studies for this review, we searched both the PubMed and Google Scholar databases for endometriosis and lncRNA combined with the following keywords: mechanism of action, sponging, ceRNA networks, chromatin, biomarkers, genome-wide, microenvironment, and infertility. We concentrated on original research papers, excluding literature reviews, pre-prints, meeting abstracts, and articles that were not in English and then assessed the remaining publications for this review.

## 3. Evidence for the Role of lncRNAs in Endometriosis

The evidence for the role of lncRNAs in the pathogenesis of endometriosis has been largely uncovered by using high-throughput screening technologies to detect differences between control and disease cohorts from either patient samples or animal models. Selected lncRNAs identified using these approaches were then subjected to further validation experiments to confirm their role. Below, we discuss the current evidence, most of which is derived from genome-wide genomic or transcriptomic studies.

### 3.1. Genetic Evidence for lncRNA Involvement in Endometriosis

Genome-wide association studies (GWAS) have been conducted for many diseases including endometriosis [24,25]. This powerful approach surveys single-nucleotide polymorphisms (SNPs) throughout the genome in a large cohort of individuals to identify variants and genomic regions associated with an increased chance of developing the disease. SNPs identified in GWAS studies often occur in intergenic regions, and therefore, it has been speculated that they may affect the regulation of nearby genes [26]. One mechanism by which this could work is via changing the sequence of an lncRNA and, thereby, affecting its regulatory function [27].

Genome-wide DNA sequencing technology has identified genetic variations in lncRNA loci that may affect the pathogenesis of endometriosis. The rs10965235 SNP located on chromosome 9p21.3 is associated with severe endometriosis in a Korean patient cohort and lies within the *CDKN2B-AS* gene locus [28]. This indicates that disruption of lncRNA function by the SNP is a possible mechanism through which this variant may predispose patients to endometriosis. The rs3820282 SNP located within a *WNT4* intron on chromosome 1p36.12 is associated with endometriosis and appears to affect an enhancer–promoter interaction resulting in the downregulation of *LINC00339* and upregulation of *CDC42* [29]. This indicates another possible mechanism for lncRNA regulation, whereby dysregulation of *CDC42* due to the risk of an SNP affecting enhancer competition with the *LINC00339* promoter leads to predisposition to endometriosis. Recently, genetic variations at the rs1838169 and rs17720428 SNPs sites on chromosome 12q13.3 in *HOTAIR* have been shown to be frequently detected in patients with endometriosis [30]. These variants appear to increase the stability of the lncRNA, resulting in reduced levels of *HOXD10* and *HOXA5* transcripts regulated by *HOTAIR*. A variant of the rs591291 SNP located on chromosome 11q13.1 in the promoter region of *MALAT1* was associated with an increase in endometriosis risk in a Chinese population, indicating that a change in the *MALAT1* expression level may affect endometriosis risk, although this was not examined in this study [31]. Finally, the rs710886 SNP in *PCAT1* has been associated with increased risk of developing endometriosis [32]. This SNP appears to disrupt sponging by *PCAT1* of *miR-145*, affecting the expression of *FASCIN1*, *SOX2*, *MSI2*, *SERPINE1*, and *JAM-A*, and the proliferative and invasive ability of endometriosis stem cells. Together, these studies indicate that genetic variants associated with endometriosis may predispose patients to the disease by disrupting the lncRNA gene regulatory function via diverse mechanisms.

### 3.2. Transcriptional Evidence for lncRNA Involvement in Endometriosis

To date, there have been around 14 genome-wide profiling transcriptome studies of human patient samples and animal models for endometriosis (Table 1). These studies are mainly descriptive in nature, with experimental validation limited to a few selected candidates. These studies often include in silico analysis examining the relationship between differentially expressed protein coding and lncRNA transcripts. Further bioinformatic approaches, such as gene ontology analysis, are then often used to predict the relevance of the identified targets to biological pathways involved in the pathogenesis of endometriosis. Initial studies were mostly conducted using microarray platforms to assess the expression of lncRNAs and mRNAs. Using a human lncRNA expression microarray, Sun et al. [33] were the first to assess genome-wide the relationship between lncRNA and mRNA expression in ovarian endometriosis lesions compared to paired autologous eutopic endometrial samples. They identified 948 lncRNAs and 4088 mRNAs as differentially expressed in the study cohort and validated the differential expression of the top 10 lncRNA candidates using qRT-PCR. Based on co-expression with mRNAs, lncRNAs in this study were predicted to take part in tissue adhesion, angiogenesis, estrogen production, and immune response, all processes known to be associated with the pathogenesis of endometriosis. Co-expression and genomic proximity were then used to predict 49 *cis*-regulating lncRNAs and their protein coding targets, while *trans*-regulating lncRNAs were predicted by co-expression with transcription factors and network analysis. This indicated that the top candidates were in a network with MYC, CTCF, and E2F4. Another study from Cai et al. (2019) used microarrays to profile lncRNA and mRNA expression in an endometriosis rat model, resulting in the identification of 115 upregulated and 51 downregulated lncRNAs together with 182 differentially expressed protein coding mRNA transcripts [34]. Co-expression analysis revealed five lncRNAs (*LOC102551276*, *NONRTT006252*, *LOC103691820*, *LOC102546604,* and *NONRATT003997*) that show a similar expression pattern to four protein coding mRNAs (*Adamts7*, *P2ry6*, *Dlx3,* and *TP53*), indicating a possible functional relationship in endometriosis.

Using a transcriptome array, Wang et al. [35] investigated the expression profile of lncRNAs in serum and tissue samples from patients with and without endometriosis. Following qRT-PCR validation of differentially expressed lncRNAs, they identified a combination of five circulating lncRNAs (*NR_038395, NR_038452, ENST00000482343, ENST00000544649,* and *ENST00000393610*) that they proposed could act as non-invasive biomarkers for the disease.

In the last few years, high-throughput RNA sequencing technology has surpassed microarrays as the preferred genome-wide technology for the identification of differentially expressed lncRNAs in endometriosis. Unlike microarrays, RNA sequencing is not biased by prior knowledge, enabling the discovery and characterization of lncRNAs that may play a role in the disease. A number of RNA sequencing studies have now been conducted comparing tissues from either patients or endometriosis animal models (Table 1). For example, using RNA sequencing, Cui et al. identified 86 differentially expressed lncRNAs and 1228 differentially expressed mRNAs in patients with ovarian endometriosis lesions compared to eutopic endometrial controls [36]. Pathway and gene ontology analysis showed that differentially expressed lncRNAs were involved in the regulation of cell proliferation, adhesion, migration, steroidogenesis, and angiogenesis, all processes implicated in endometriosis lesion formation and survival at ectopic sites of implantation.

**Table 1:** Genome-wide studies that identified differentially expressed lncRNAs in endometriosis.

Method (Reference)	Cohort (Tissue Type)	Validation (Type; Cohort; lncRNAs)	Predicted Function in Endometriosis	Limitations
Expression Microarray [33]	*n* = 8 (paired ovarian: 4 eutopic and 4 ectopic tissues)	RT-qPCR *n* = 42 (paired ovarian: 21 eutopic and 21 ectopic tissues) *CHL1-AS2*, *MGC24103*, *XLOC_007433, HOXA11-AS, KLP1, LOC100505776, XLOC_012981, LIMS3-LOC4408, LOC389906*	*Cis* and *trans* regulation of protein coding genes	1, 6
Expression Microarray [37]	*n* = 6 (3 eutopic tissues of women with EM of undefined entity and 3 eutopic tissues of women without EM)	RT-qPCR *n* = 68 (40 eutopic tissues of women with EM of undefined entity and 28 eutopic tissues of women without EM) *RP11-369C8.1, RP11-432J24.5, AC068282.3, GBP1P1, SNHG1, AC002454.1, AC007246.3, FTX*	Cell cycle regulation and immune response	1, 2, 4, 6
Expression Microarray [35]	Serum: *n* = 20 (10 women with peritoneal and/or ovarian EM and 10 women without EM) Tissue: *n* = 15 (paired peritoneal and/or ovarian: 5 eutopic and 5 ectopic EM patients and 5 eutopic tissues of women without EM	RT-qPCR Serum: *n* = 110 (59 women with peritoneal and/or ovarian EM and 51 women without EM) Tissue: qPCR *n* = 24 (paired peritoneal and/or ovarian: 9 eutopic and 9 ectopic tissues of EM patients and 6 eutopic tissues of women without EM) DE lncRNAs 16	Combination of NR_038395, NR_038452, ENST00000482343, ENST00000544649, and ENST00000393610 suggested as a non-invasive diagnosis marker	1, 2 (because of pooling)
Expression microarray [38]	*n* = 8 (paired ovarian: 4 eutopic and 4 ectopic tissues of EM patients)	RT-qPCR *n* = 87 (paired ovarian: 30 eutopic and 30 ectopic tissues of EM patients and 27 eutopic tissues of women without EM)) *CHL1-AS2*	CCDC144NL-AS1 expression was upregulated in ectopic tissues compared to eutopic and control endometrial tissues	1
Re-analysis of existing microarray data [39]	*n* = 18 GSE120103 (eutopic tissue from 9 fertile and 9 infertile women with ovarian EM)	Validation of 14 hub mRNAs using GSE26787 (5 fertile and 5 infertile women with unknown EM status)	Identification of putative infertility-associated lncRNAs *LOC390705* and *LOC100505854*	1, 3, 6
Re-analysis of existing microarray data [40]	GSE7305 (paired ovarian: 10 eutopic and 10 ectopic tissues of EM patients and 10 eutopic tissues of women without EM) GSE7846 (HEECs of eutopic tissues of 5 EM patients with ovarian EM and HEECs of eutopic tissues of 5 women without EM), GSE29981 (LMD epithelial cells of 20 women without EM) E-MTAB-694 (paired peritoneal: 18 eutopic and 18 ectopic tissues of EM patients and 17 eutopic tissues of women without EM)	No validation	Proposed cell cycle regulatory functions for *LINC01279*	2, 3, 4, 5
RNA-Seq [41]	*n* = 16 (8 eutopic tissues of women with EM of undefined entity and 8 eutopic tissues of women without EM)	No validation	Predicted oxidative stress and endometrial receptivity regulatory functions	1, 2, 3, 4, 6
RNA-Seq [36]	*n* = 10 (5 eutopic tissues of patients with ovarian EM and 5 eutopic tissues of women without EM)	RT-qPCR *n* = 24 (12 eutopic tissues of patients with ovarian EM and 12 eutopic tissues of women without EM) *USP46-AS1, RP11-1143G9.4, RP11-217B1.2, AC004951.6, RP11-182J1.12*	Predicted proliferation, adhesion, migration, invasion, and angiogenesis regulatory functions	1, 4, 6
RNA-Seq [42]	*n* = 9 (paired ovarian: 3 eutopic and 3 ectopic tissues of EM patients and 3 eutopic tissues of women without EM)	RT-qPCR *n* = 45 (paired:15 eutopic and 15 ectopic tissues of EM patients with undefined entity and 15 eutopic tissues of women without EM) *PRKAR2B*, *CLEC2D*	Predicted angiogenesis, cell adhesion, cell migration, immune response, inflammatory response, NF-κB signaling, regulatory functions	1, 2, 4
Re-analysis of existing RNA-Seq and expression array datasets [43]	GSE105764 (paired ovarian: 8 eutopic and 8 ectopic tissues of EM patients) GSE121406 (paired ovarian: FACS sorted stromal cells of 4 eutopic and 4 ectopic tissues of EM patients) GSE105765 (same as GSE105764)	in silico validation by microarray GSE124010 (3 ectopic tissues of patients with EM of undefined entity and 3 eutopic tissues of women without EM) GSE86534 (paired ovarian: 4 eutopic and 4 ectopic tissues of patients with EM)	Predicted function in regulation of inflammation and prediction of sponging *LINC01018* and *SMIM25* functions for *hsa-miR-182-5p* in the regulation of *CHL1* protein coding gene to promote endometriosis	1, 4 (validation study), 5, 6
Re-analysis of existing RNA-Seq datasets [44]	*n* = 28 (paired ovarian: 14 eutopic and 14 ectopic tissues of infertile EM patients) GSE105764 (paired ovarian: 8 eutopic and 8 ectopic tissues of EM patients) GSE105765 (same patients as in GSE105764) GSE25628 (unpaired DIE: 8 eutopic and 8 ectopic tissues of EM patients and 6 eutopic tissues of women without EM)	No validation	Construction of a competitive endogenous (ce) RNA network promoting growth and death of endometrial stroma cells. CDK1 and PCNA proposed as treatment targets for endometriosis- associated infertility	1, 3, 5
RNA-Seq [45]	*n* = 12 (paired ovarian: 6 eutopic and 6 ectopic tissues of EM patients)	RT-qPCR *n* = 60 (paired ovarian: 30 eutopic and 30 ectopic tissues of EM patients) *MIR202HG, LINC00261, UCA1, GAGA2-AS1*	Immunity, inflammation	1, 6
Re-analysis of existing RNA-Seq data [46]	GSE105764 and GSE105765 include same patients *n* = 16 (paired ovarian: 8 eutopic and 8 ectopic tissues of EM patients)	No validation	*LncRNAs:H19, GS1-358P8.4,* and *RP11-96D1.10* strongly associated with ovarian endometriosis	1, 3, 6
Animal Studies				
Expression microarray [34]	Rat *n* = 35 (EM uterine tissue EM = 13, adipose tissue control = 8, blank = 14)	RT-qPCR; NONRATT003997; gi|672033904|ref|, XR_589853.1|; NONRATT006252; gi|672027621|ref|; XR_592747.1|; gi|672045999|ref|; XR_591544.1|	Regulation of endometrial receptivity	

EM, endometriosis; HEECs, human endometrial endothelial cells; LMD, laser microdissection; DIE, deep infiltrating endometriosis. 1, small sample size (less than 30 per group); 2, no comprehensive clinical information (i.e., American Fertility Society (rAFS) disease stage, lesion entities, menstrual cycle phase); 3, no validation in an adequate independent cohort; 4, EM-free controls are not appropriate (i.e., CIN patients, no laparoscopic proof); 5, combining heterogeneous datasets (i.e., different lesion entities, cycle phases, stages, cell types); 6, not all relevant tissues analyzed (i.e., eutopic tissues of EM-free controls, eutopic and ectopic tissues of EM patients).

In spite of the advantages listed above, RNA sequencing studies have some limitations. Due to their high cost, they are often limited to a small number of samples and lack extensive validation. However, they can form the basis for further studies that perform functional validation of candidate genes and investigate their value as diagnostic and prognostic markers in endometriosis. These validation studies usually have cohorts with a larger sample size and may include functional experiments that attempt to uncover the molecular mechanism of lncRNA action (Table S1). For example, AFAP1-AS1 was identified as one of the most differentially expressed lncRNAs in the microarray study of Sun et al. [33]. In a new study, Lin et al. [47] could confirm and extend these findings in a cohort of *n* = 36 patients. They show that *AFAP1-AS1* is overexpressed in ectopic endometrium of women with endometriosis (*n* = 18), compared to paired eutopic endometriosis tissue (*n* = 18) and normal endometrium of women without the disease (*n* = 10). In vitro gene targeting assays in primary human endometriotic stroma cells (ESCs) indicated that this lncRNA regulates epithelial–mesenchymal transition (EMT) in endometriosis by regulating transcription of the EMT-related transcription factor ZEB1. Furthermore, experiments in a xenograft mouse model indicated that AFAP1-AS1 was required for the growth of ectopic tissue. In this case, a shRNA knockdown of AFAP1-AS1 in the Ishikawa endometrial cancer cell line led to reduction of the subcutaneous tumor size, compared to animals injected with a non-targeting shRNA-transfected cell line [47].

Liu et al. analyzed the value of lncRNA H19 expression as an endometriosis biomarker [48]. They found that H19 expression in the both the ectopic and eutopic endometrium of endometriosis patients was significantly higher than in the normal endometrium. Overexpression of H19 lncRNA in endometriosis lesions was associated with infertility, recurrence of disease, bilateral ovarian lesions, an increased CA125 level and with progression in the revised American Fertility Society (rAFS) disease stage. Further multivariate logistic regression analysis showed that H19 overexpression in endometriosis lesions is a prognostic factor for endometriosis recurrence.

**Figure 2:**
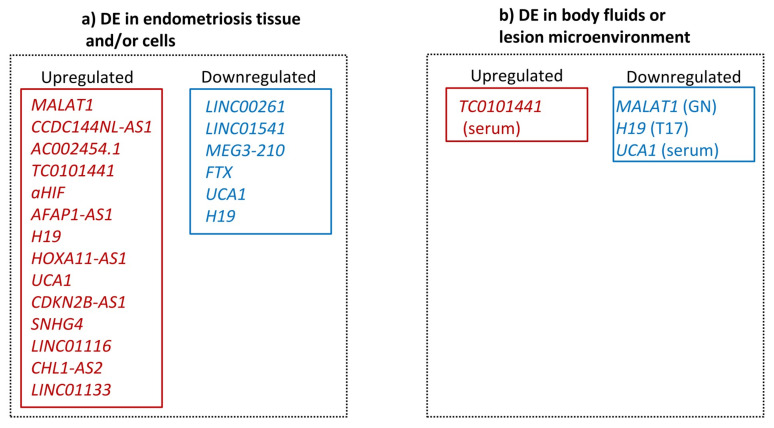
Differentially expressed (DE) lncRNAs in endometriosis based on validation studies: (**a**) Up- and downregulated lncRNAs in endometriosis tissues and/or cells (**b**) in body fluids or lesion microenvironment.

In summary, recently there has been an effort by a number of studies using genome-wide high-throughput technologies to identify differentially expressed lncRNAs in endometriosis not only for their potential clinical application as diagnostic or prognostic biomarkers of the disease, but also in order to gain insights into the pathogenesis of the disease (Table 1). The lncRNAs where further work has been done to validate a role in endometriosis are listed in Table S1 and summarized in Figure 2.

### 3.3. Critical Assessment of the Evidence for the Role of lncRNAs in Endometriosis

In the previous sections, and in Table 1 and Table S1, we summarized the evidence for a role for lncRNAs candidates in endometriosis derived from genomic and transcriptomic studies. However, the strengths and weaknesses of these studies should be taken into account when assessing the role of individual lncRNAs in the pathology of the disease.

We have assessed the studies listed in Table 1 and Table S1 and noted common limitations, namely small sample size, incomplete clinical information, no validation in an independent cohort, inappropriate controls, or failure to examine all relevant tissues. A limitation that can apply in particular to re-analysis of published datasets is that differences in the type of tissue collected, such as lesion type, menstrual cycle stage, or the type of controls, used may limit the validity of comparisons between studies. Following assessment of all the studies in Table 1 and Table S1, we found that 38.5% (5/13) of the transcriptome studies had no validation in an adequate independent cohort and that 23.1% (3/13) compared heterogeneous datasets that containing either different lesion types, cell types, or disease stages, and/or samples from different menstrual cycle phases. In total, 81.1% (30/37) of all studies analyzing lncRNA expression in human tissues had a small sample size (defined as less than 30 per group), and 45.9% (17/37) did not provide comprehensive clinical information regarding the lesion type, rAFS stage, or menstrual cycle phase. Around half of the studies (19/37) had inappropriate endometriosis-free controls, in that they included cervical intraepithelial neoplasia (CIN) patients or had no laparoscopic proof that the controls were endometriosis free. Finally, 45.9% (17/37) of the studies did not evaluate all the relevant tissue types, which are eutopic tissues of endometriosis-free controls and eutopic and ectopic tissues from endometriosis patients. Therefore, while these genome-wide studies can be valuable in detecting lncRNAs that may be novel players in endometriosis pathogenesis, careful validation studies are required, as well as mechanistic studies to understand how they may function.

## 4. Functional Evidence for the Mechanism of lncRNA Action in Endometriosis

The diverse range of mechanisms for lncRNA action described above show what is possible, but so far, only a subset of these mechanisms have been described in the context of endometriosis. Based on the current knowledge, the mechanism of lncRNA action in endometriosis can be divided into the following groups: (i) lncRNAs that recruit and target chromatin remodeling or transcriptional regulatory factors, (ii) lncRNAs with miRNA sponging functions, and (iii) lncRNAs that modulate cellular signaling pathways.

### 4.1. Chromatin Remodeling and Transcriptional Control by lncRNAs in Endometriosis

The HOTAIR lncRNA is known as a critical regulator of HOX gene expression. Maintaining appropriate expression of genes within the HOX-gene network has been shown to be critical for endometrial homeostasis during embryonic implantation, and alterations in HOXA10 and HOXA11 expression have been associated with endometriosis-associated infertility [49,50]. Mechanistically, *HOTAIR* interacts with the PRC2 or REST/CoREST chromatin remodeling complex to guide the recruitment of H3K27 tri-methylation or H3K4-demethylation at target gene loci resulting in target gene silencing [51]. As described above, it has been shown that genetic variations at SNPs sites in *HOTAIR* are relatively frequently detected in patients with endometriosis [30]. These genetic changes alter the thermostability of mature *HOTAIR* leading to epigenetic silencing of *HOXD10* and *HOXA5* genes and are associated with more advanced endometriosis [30].

Overexpression of the lncRNA *AFAP1-AS1* has been shown to be associated with ovarian endometriosis [33]. In another example of direct transcriptional regulation by an lncRNA in endometriosis, *AFAP1-AS1* directly activates expression of EMT-related transcription factor ZEB1 in vitro [47]. ZEB1 has been previously shown to be involved in 17β-estradiol-induced EMT in endometriosis [52], indicating a possible mechanism by which *AFAP1-AS1* could affect endometriosis.

### 4.2. MiRNA Sponging by LncRNAs in Endometriosis

A growing body of evidence indicates that lncRNAs can act as miRNA sponges in endometriosis (summarized in Table 2). In these cases, a binding site for the miRNA is present in both the lncRNA and the targeted protein-coding gene, and there is a correlation between expression of the lncRNA and the protein-coding gene. In the first reported example of sponging in endometriosis, decreased levels of the H19 lncRNA was shown to be associated with an increase in *let-7* miRNA activity, which inhibits *IGF1R* expression resulting in the reduced proliferation of endometrial stroma cells [53]. These results suggested that the *H19*/*let7*/IGF1R pathway may contribute to impaired endometrial receptivity in women suffering from the disease. *H19* was also shown to regulate cell proliferation and invasion of ectopic endometrial cells by increasing ITGB3 expression via sponging of *miR-124-3p* [54]. *H19* has also been implicated in the impaired immune responses of women with the disease, by acting as a sponge for *miR-342-3p*, which regulates the IER3 pathway. This pathway has been implicated in Th-17 cell differentiation and endometrial stroma cell proliferation in ectopic sites in women with the disease [55]. Another lncRNA that has been shown to act as a sponge in endometriosis is *CDKN2B-AS1*, which acts as a regulator of AKT3 expression by sponging *miR-424-5p* in an in vitro model of ovarian endometriosis [56]. In another example, *LINC01116* promoted the proliferation and migration of endometrial stroma cells by targeting FOXP1 via sponging of *miR-9-5p*, thereby promoting endometriosis lesion formation and growth [57]. *MALAT1* was identified as a sponge of *miR-200c* involved in the regulation of endometriosis stoma cell proliferation and migration by promoting ZEB1 and ZEB2 expression in women with the disease [58]. This regulation is not restricted to *miR-200c* and may include the entire *miR-200* family, consisting of *miR-200a*, *miR-200b*, *miR-200c*, *miR-141*, and *miR-429* [59]. In addition to these examples, other lncRNAs have also been implicated in endometriosis via their role as sponges for miRNAs, as summarized in Table 2.

### 4.3. LncRNAs Modulating Cellular Signaling Pathways in Endometriosis

Cell signaling pathways have a pivotal role in the regulation of a variety of cellular processes in response to intracellular or extracellular stimuli. The regulation of components of a signaling pathway by lncRNAs can be direct or indirect and result in functional changes in the signaling cascades. Direct regulation can be achieved by direct binding of the lncRNA to signaling proteins leading to changes in either their free cellular levels or their activity. We define indirect regulation as cases where no direct lncRNA binding to signaling molecules has been shown and where the lncRNA is thought to alter the transcription of genes associated with the signaling pathway resulting in an altered cellular response. In Table 3, we summarize currently known cases where a lncRNA directly or indirectly affects a signaling pathway in endometriosis.

In an example of direct regulation of a signaling pathway in endometriosis, the lncRNA *MEG3-210* has been shown to regulate endometriosis stromal cell migration, invasion, and apoptosis through the p38 MAPK and PKA/SERCA2 signaling pathways. In this case, *MEG3-210* directly interacts with Galectin-1 in vitro and affects the growth of endometriotic lesions in vivo in a murine model of the disease [64]. Mechanistically, *MEG3-210* titrates away the cellular levels of Galectin-1, preventing its action on the p38 MAPK and PKA/SERCA2 signaling cascades in endometrial stromal cells. In endometriosis, the levels of *MEG3-210* are downregulated and the levels of free Galectin-1 are upregulated, which is associated with the subsequent activation of p38 MAPK signaling–mediated phosphorylation of ATF2. Activated ATF2 increases the expression of BCL-2 and MMP contributing to the anti-apoptotic, pro-migratory, and invasive phenotype of endometriosis cells. Simultaneously, *MEG3-210* downregulation leads to suppression of the PKA/SERCA2 signaling cascade [64].

**Figure 3:**
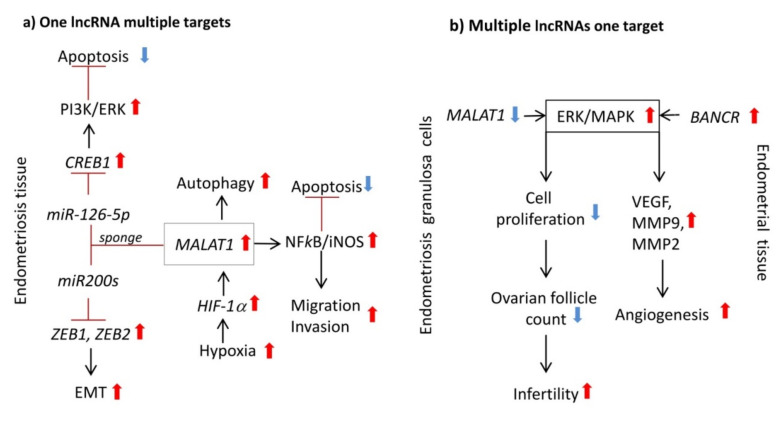
(**a**) *MALAT1* was identified as a sponge of *miR-200c*. This regulation is not restricted to *miR-200c* and might include the entire *miR-200* family (*miR-200s*), consisting of *miR-200a*, *miR-200b*, *miR-200c*, *miR-141*, and *miR-429*. Upregulation of *MALAT1* in women with endometriosis leads to enhanced sponging of *miR200s* and promotes zinc finger E-box binding homeobox transcription factor 1 (ZEB1) and ZEB2 expression leading to higher EMT. In HESCs, the lncRNA *MALAT1* directly interacts with *miR-126-5p*, which regulates cAMP responsive element-binding protein (CREB1) expression. Upregulation of *MALAT1* inhibits apoptosis probably via activation of the PI3K–AKT pathway through the *miR-126-5p*–CREB1 axis. *MALAT1* lncRNA can also lead to reduced apoptosis in HESCs through the upregulation of the NFkB/iNOS signaling pathway activity, which also enhances migration and invasion of cells. In cultured primary endometrial stromal cells, *MALAT1* leads to upregulation of hypoxia-induced autophagy. In this signaling cascade, regulation of *MALAT1* expression is under the control of the HIF1α transcription factor. (**b**) In granulosa cells (GCs) of women with endometriosis, significant downregulation of *MALAT1* expression was reported. *MALAT1* knockdown induced an increase in phosphorylated ERK1/2 (p-ERK1/2) that was associated with altered follicle count, due to impaired cell proliferation resulting from ERK/MAPK-dependent activation of p21/p53 cell cycle arrest. In an autograft transplantation rat model of endometriosis, inhibition of the lncRNA *BANCR* led to a decrease in ectopic tissue volume associated with a significant reduction in serum levels of *VEGF*, *MMP-2*, and *MMP-9, ERK*, and *MAPK* mRNA and in phosphorylated ERK and MAPK protein levels in tissues. HESCs: human endometrial stromal cells.

In an example of indirect regulation of signaling pathway, knockdown of *MALAT1* lncRNA in endometriosis cells leads to enhanced cell death, reduced migration and invasion associated with activation of Caspase-3, and downregulation of MMP-9 and the NFkB/iNOS signaling pathway (Figure 3a) [65]. In contrast to endometriosis tissue where *MALAT1* is overexpressed, in the granulosa cells of women with endometriosis *MALAT1* expression is reduced [66]. This is associated with a reduced follicle count, due to impaired cell proliferation resulting from ERK/MAPK-dependent p21/p53 cell cycle arrest. This implicates altered expression of *MALAT1* in endometriosis-related infertility. In cultured primary endometrial stromal cells, depletion of *MALAT1* by siRNA knockdown results in the suppression of hypoxia-induced autophagy, as indicated by a reduction in the expression of autophagy markers Beclin-1 and LC3-II [67]. In this signaling cascade, expression of *MALAT1* is regulated by the HIF1α transcription factor, known to be overexpressed in endometriosis lesions and to regulate multiple gene targets in response to hypoxia (Figure 3a) (reviewed in [76]).

In another example, the most downregulated lncRNA in ectopic tissue of women with ovarian endometriosis was *LINC01541* [33], which has been shown respond to levels of estradiol [68]. A gene-targeting in vitro study in human endometrial stromal cells showed that the cellular levels of *LINC01541* affect the activity of WNT/β-catenin, pro- and anti-apoptotic signaling regulators Caspase-3 and BCL2 and the levels of VEGFA production [68].

The presence of extracellular lncRNA in exosomes or microvesicles raises the possibility that these lncRNAs may be able to serve as extracellular signals for cell signaling regulation in endometriosis. This was supported by reports that exosomal lncRNAs promote angiogenesis in endometriosis [77]. Based on an in vitro co-culture model and patient serum analysis, the authors developed a novel mechanistic model explaining how endometriosis stromal cells of the lesion induce angiogenesis. They suggested that these cells in an ectopic environment can produce exosomes that are enriched in *aHIF* lncRNA, a pro-angiogenic lncRNA, highly expressed in ectopic endometrial stromal tissue. These *aHIF*-rich exosomes are then taken up by recipient macrovascular cells, where they cause upregulation of the angiogenesis-related genes VEGF-A, VEGF-D, and bFGF.

Endometriosis is an estrogen-dependent disease, and therefore, lncRNAs involved in the estrogen pathway or those targeted by estrogen signaling could play a role in the disease. The lncRNA *H19* is positively regulated by estrogen, and its expression in the endometrium increases during the proliferative stage of the menstrual cycle [78,79]. *H19* has been shown regulate several pathways that are relevant in endometriosis including IGF1R, ITGB3, IER3, and ACTA2 [53,54,55,80]. Another lncRNA, *steroid receptor RNA activator 1* (*SRA1*) lncRNA, has been reported to act in concert with SRA1 to regulate the expression of estrogen receptors by affecting alternative splicing, and thereby the growth of stromal cells in ovarian endometriosis [81]. These examples illustrate how lncRNAs could play a role in endometriosis via the estrogen pathway or as targets of estrogen regulation.

Conceptually, the mechanism of lncRNAs’ action in endometriosis may involve influencing cellular pathways, where one lncRNA may regulate several targets using different molecular strategies (Figure 3a) or one targeted signaling pathway may be affected by multiple lncRNAs (Figure 3b). To better understand this complexity, integrative in silico and experimental analyses interrogating the molecular networks in endometriosis cells need to be applied. This approach should help to dissect the relationship between lncRNA, mRNA, miRNA expression, genetic- and epigenetic-driven chromatin remodeling, and signal transduction activity and to enable lncRNA target identification. The use of such in silico bioinformatics algorithms for cellular network construction in endometriosis has already been attempted by several studies [41,43,46]. For example, Wang et al. [41] constructed an lncRNA–miRNA–mRNA network, revealing lncRNAs that act as competing endogenous RNAs (ceRNA), the miRNAs they sponge, and the target genes that were involved in regulating endometrial receptivity in endometriosis. Moreover, Jiang et al. [43] identified the lncRNAs (*SMIM25*, *LINC01018*), miRNA (*miR-182-5p*), and mRNA (*CHLI*) as part of a ceRNA network implicated in the regulation of immune responses in endometriosis. These in silico studies may be valuable to predict how lncRNAs may function in endometriosis, but experimental validation is required in order for these predictions to be confirmed.

## 5. Summary and Perspectives

**Figure 4:**
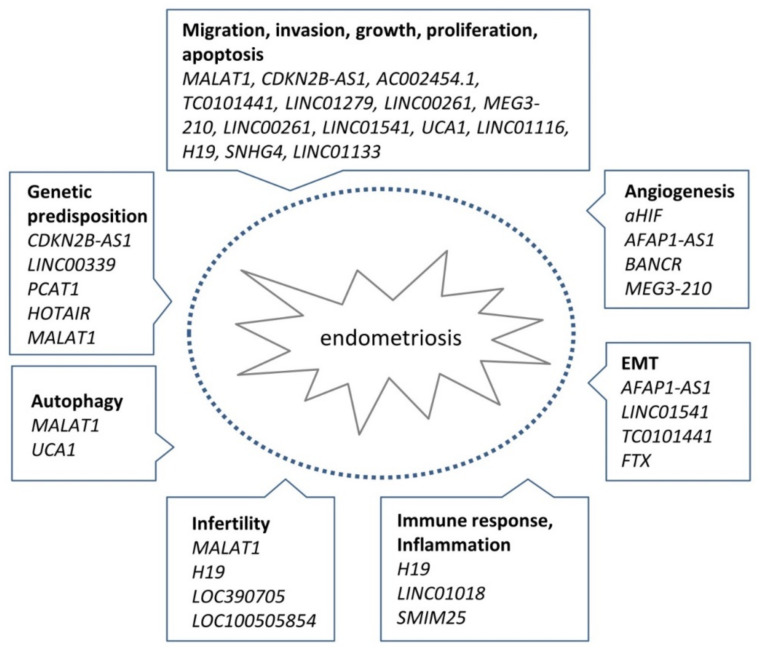
Phenotypical changes caused by altered expression of lncRNAs in endometriosis. Altered expression of lncRNAs in endometriosis is involved in the regulation of numerous processes known to be associated with the pathogenesis of the disease. These processes include EMT, endometriosis cell stemness, angiogenesis, lesion establishment and growth, endometriosis cell survival, proliferation and invasion, oxidative stress, autophagy, and endometrial receptivity.

In recent years, a growing number of studies have implicated the abnormal expression of specific lncRNAs with various aspects of endometriosis pathogenesis. This altered expression may be due to genetic predisposition or an unknown environmental trigger and affect pathogenetic processes including EMT, endometriosis cell stemness, angiogenesis, lesion establishment and growth, endometriosis cell survival, proliferation and invasion, oxidative stress, autophagy, and endometrial receptivity (summarized in Figure 4). Evidence for the role of lncRNAs has mainly come from large-scale transcriptome studies comparing normal and diseased tissue, followed by validation studies of a small number of candidate lncRNAs. These studies can be valuable for uncovering potential novel players in endometriosis but often have weaknesses that should be considered including small sample size, incomplete clinical information on patient samples, and inappropriate controls (Table 1, Table S1). A general weakness of these studies is that they do not assess the complexity of the disease to take into account different lesion types, the stage or severity of the disease, or the effect of hormone status or age of the patient. The vast majority of lncRNAs revealed in these studies remain unvalidated by an independent method or by functional studies. One challenge for future work is to prioritize lncRNAs identified in these studies for validation and then conduct further functional studies to determine their role in endometriosis and their possible use as biomarkers or targets for treatment.

One avenue of endometriosis research that should be pursued in the future is the connection between genetic variants and abnormal lncRNA expression that affects aspects of endometriosis pathogenesis. The majority of disease-associated SNPs in GWAS studies, including those investigating endometriosis, are in non-coding regions. The hypothesis that these SNPs may affect lncRNA function is supported by a number of studies in endometriosis, with SNPs in or near lncRNAs including *HOTAIR, MALAT1, CDKN2B-AS, PCAT1,* and *LINC00339* affecting their expression and regulation of genes and the endometriosis phenotype [28,29,30,31,32]. The possibility that other non-coding SNPs associated with endometriosis may also affect the disease via lncRNAs should be investigated.

In vitro cell culture models and in vivo animal models are valuable systems that enable the mechanism of action and function of individual lncRNAs to be investigated experimentally. However, a weakness of some studies investigating endometriosis, including those investigating the role of lncRNAs, is that the available in vitro and in vivo models may not always represent all aspects of the disease accurately. Therefore, it should be a priority for the field to continue to strive for improved models to investigate the disease.

To conclude, genome-wide genomic and transcriptomic studies have implicated numerous lncRNAs in a wide range of diseases, including in endometriosis. The challenge remains to distinguish the lncRNAs that play a relevant role in the disease from those that are merely associated with the transcriptional changes in the disease and to functionally show their role.

## Figures and Tables

**Table 2:** LncRNAs involved in endometriosis as molecular sponges of miRNAs.

lncRNA (Reference)	Sponged miRNA	Target mRNA (Pathway) in Endometriosis
*H19* [53]	*Let-7; miR-125-3p; miR-342-3p; miR-216a-5p*	*IGF1R*; *ITGB3*; *IER3*; *ACTA2*
*CDKN2B-AS1* [56]	*miR-424-5p*	*AKT3*
*LINC01541* [60]	*miR-506-3p*	*WIF1* (Wnt/β-catenin)
*LINC01116* [57]	*miR-9-5p*	*FOXP1*
*SNHG4* [61]	*miR-148a-3p*	*c-Met*
*LINC01018* [43]	*miR-182-5p*	*CHLI* (inflammatory)
*SMIM25* [43]	*miR-182-5p*	*CHLI* (inflammatory)
*MALAT1* [62]	*miR-126-5p; miR200s; miR200c*	*CREB1* (PI3K/AKT); *ZEB1*, *ZEB2*, *VIM* (EMT); *ZEB1*, *ZEB2*; *CDH2* (EMT)
*LINC00261* [63]	*miR-132-3p*	*BCL2L11*
*PCAT1* [32]	*miR-145*	*FASCIN1*, *SOX2*, *MSI2*, *SERPIN*

**Table 3:** Mechanisms of cell signaling regulation via lncRNAs in endometriosis.

lncRNA (Reference)	Model System	Signaling Molecules	Signaling Pathways	Type of Regulation	Function in Endometriosis
*MEG3-210* [64]	Primary HESC, HEEC EM mouse model	Galectin-1	P38 MAPK, PKA/SERCA2	direct	Regulation of migration, invasion and apoptosis, lesion growth and vascularization
*MALAT1* [65]	EMs cells	Caspase-3, MMP-9	NFkB/iNOS	Indirect	Regulation of apoptosis, migration, invasion
*MALAT1* [66]	Granulosa cells (KGN cell line)	p21, p53, CDK1	ERK/MAPK	Indirect	Regulation of cell proliferation, ovarian follicle count, infertility
*MALAT1* [67]	HESC	HIF-1α, LC3-II, beclin1	-	Indirect	Regulation of hypoxia-induced pro-survival and autophagy
*LINC01541* [68]	HESC	β-Catenin, VEGFA, BCL2, caspase-3	WNT/β-Catenin	Indirect	Regulates EMT, migration, invasion, survival, and angiogenesis
*LINC01541* [69]	12Z epithelial endometriosis cell line	p21, cyclin A	TESK1/Cofilin	Indirect	Regulation of cell proliferation and invasion
*FTX* [70]	EESC (ectopic endometrial stromal cells), HESC (normal)	E-Cadherin, N-cadherin, ZEB1, vimentin	PI3K/AKT	Indirect	Regulates EMT and cell cycle
*BANCR* [71]	Rat model of EM, ectopic tissue, and serum	VEGF, MMP-9, MMP-2	MAPK/ERK	Indirect	Regulation of angiogenesis
*UCA1* [72]	HESC	IC3, VMP1	-	Indirect	Regulation of autophagy and apoptosis
*AC002454.1* [73]	EESC	CDK6	-	Indirect	Regulation of cell migration and invasion
*CCDC144NL-AS1* [74]	HESC	Vimentin, MMP-9	-	Indirect	Regulation of cell migration and invasion
*TC0101441* [75]	ECSC	N-Cadherin, SNAIL, SLUG, TCF8/ZEB1	-	Indirect	Promotes endometriosis cyst stromal cell (ECSC) migration and invasion

## Data Availability

Not applicable.

## Supplementary Materials

The following are available online at https://www.mdpi.com/article/10.3390/ijms222111425/s1.
