# Supplementary material for: The Role of Long Non-Coding RNAs in Endometriosis
Source: Int J Mol Sci. 2021 Oct 22;22(21):11425. doi: 10.3390/ijms222111425 (PMC8583837; doi:10.3390/ijms222111425)
Supplement: Supplementary file 1 [file ijms-22-11425-s001.zip › ijms-1370281-supplementary.pdf]

Table S1. Functional studies analyzing differentially regulated lncRNAs in endometriosis using qRT-PCR.

lncRNA[Reference]	Number of samples	Source/Cycle Phase	Outcome	Limitations
UCA1 [1]	Tissue:<br>98 paired ovarian EM<br>Serum:<br>98 + 28 controls	Human<br>Tissue<br>Serum	Reduced expression in ectopic tissues compared to paired eutopic tissues.<br>Diagnostic and prognostic marker for ovarian EM (AUC=0.992; p<0.0001)<br>Marker for Recurrence in a 2- year follow-up	3
UCA1 [2]	30 eutopic (of which 15 with ovarian EM)<br>+30 controls (CIN)	Human:<br>Tissue	Higher expressed in eutopic endometrium of patients with endometriosis compared to controls.<br>Prognostic marker and therapeutic target.	2; 3; 4
MALAT1 [3]	15 ectopic tissues of undefined entity<br>+ 7 controls	Human:<br>Tissue	Higher expressed in ectopic endometrium tissue of women with EM when compared to controls.	1; 2; 3; 4
MALAT1 [4]	30 paired ovarian<br>+ 30 controls	Human:<br>Tissue<br>Proliferative	Higher expressed in ovarian EM lesions than in controls and eutopic endometrium with EM.	
MALAT1 [5]	12 ectopic tissues of undefined entity<br>+ 8 controls (CIN)	Human:<br>Tissue	Higher expressed in ectopic tissue compared to controls.	1; 2; 3; 4
MALAT1 [6]	52 patients with EM of undefined entity<br>+ 52 controls	Human:<br>Granulosa Cells	Diagnostic and prognostic marker (AUC=0.7)<br>AUC=0.8 for severe stages;<br>Reduced expression in GCs in women with EM may impair fertility.	2; 3
MALAT1 [7]	20 ectopic ovarian<br>20 eutopic EM<br>+ 20 controls	Human:<br>Tissue<br>Proliferative	Higher expressed in ectopic tissues compared to eutopic endometrium of patients with EM and to controls.	1; 3
HOXA11-AS1 [8]]	30 paired peritoneal<br>+ 15 infertile controls	Human:<br>Tissue<br>Implantation window:<br>Midsecretory (day20-23)<br>serum progesterone>10 ng/ml	Reduced expression in eutopic tissue compared to paired ectopic tissues of patients with EM. No difference in eutopic tissues between women with and without EM.	1
H19 [9]	10 eutopic tissues of undefined entity<br>+10 controls	Human:<br>Tissue	Reduced expression in eutopic endometrium of women with EM compared to controls.	1; 2; 3; 4

H19 [10]	104 eutopic and ectopic of mixed entities + 50 controls (CIN)	Human: Tissue Late Proliferative (days 11-13)	Higher expressed in the ectopic and eutopic tissues of patients with EM compared to controls. High H19 expression was positively correlated with infertility, recurrence, bilateral ovarian lesions, CA125 level, and rAFS stage. Prognostic marker for recurrence in a 2-3 year follow up.	3
H19 [11]	20 patients with ovarian EM + 16 controls	Human: Mononuclear cells of peritoneal Fluid (PF) Mice: allotransplantati on	Reduced in PF of patients with EM. mice: weight of intraperitoneal endometriotic lesions was increased in the endometriosis group after induction for Th17 polarization and H19 overexpression reversed this effect.	1; 2
MEG3-210 [12]	Tissue: 40 eutopic tissues of undefined entity +39 controls	Human : Tissue Mice	Reduced in the eutopic endometrium of women with EM compared to controls. In the endometriosis mouse model the size of subcutaneous endometriotic lesions in the sh*MEG3-210* group was larger than that in the non-targeting sh-control group.	1; 2; 3; 4
AFAP1-AS1 [13]	18 paired ovarian + 10 controls	Human: Tissue Proliferative Mice: subcutaneous Ishikawa cells	Higher expressed in ectopic endometrium compared to paired eutopic endometrium and controls. Knockdown reduced tumor size in xenograft mouse model.	1; 2; 3
SNHG4 [14]	25 paired ovarian + 20 controls	Human: Tissue Proliferative (days 11-13) Mice (n=8): Ectopic HESCs	Higher expressed in ectopic tissues compared to the paired eutopic tissue from women with EM and to the controls. In the endometriosis mouse model the volume of subcutaneous HESC lesions was reduced in the si-SNHG4 group compared to the non-targeting sh-control group.	1
LINCO1116 (TALNEC2) [15]	20 paired ovarian	Human: Tissue	Higher expressed in ectopic tissues compared to the paired eutopic tissues of women with EM.	1; 2; 4
LINC00339 [16]	8 paired peritoneal + 8 controls	Human: Tissue	Using *in situ* hybridization they found no differences between eutopic and ectopic tissues.	1
LINCO1541 [17]	18 ectopic ovarian EM (stage III+IV) + 10 controls	Human: Tissue	Reduced expression in ectopic tissues compared to controls.	1; 2; 3; 4
FTX [18]	38 eutopic of undefined entity + 20 controls	Human: Tissue	Reduced expression in eutopic tissues of women with EM compared to controls.	1; 2; 3; 4

LINC00261 [19]	20 paired EM tissues of undefined entity	Human: Tissue	Reduced expression in ectopic tissues compared to paired eutopic tissues of women with EM.	1; 2; 4
CDKN2B-AS1 [20]	21 paired ovarian + 24 controls (CIN)	Human: Tissue	Higher expressed in ectopic and eutopic tissues of women with endometriosis compared to controls.	1; 3
CHL1-AS1 and *CHL1-AS2* [21]	30 paired ovarian + 27 controls	Human: Tissue	Higher expressed in ectopic tissues compared to eutopic tissues of women with and without EM. No difference between eutopic tissues of women with and without EM.	1
aHIF [22]	Tissue: 30 ectopic ovarian EM rAFS III+IV (10 paired from the 30) + 16 controls Serum: 30 with + 16 controls	Human: Tissue Serum	Higher expressed in ectopic tissues compared to paired eutopic tissue of women with EM and to controls. Circulating serum exosomal *aHIF* was elevated in patients with EM. Serum exosomal *aHIF* levels in patients with EM was significantly correlated to *aHIF* expression in matched ectopic endometria.	1
TC0101441 [23]	Tissue: 10 paired eutopic and ectopic ovarian EM +10 controls Serum: 29 +16 controls	Human: Tissue Serum	Higher expressed in ectopic tissues compared to paired eutopic tissue of women with EM and to controls. Circulating serum exosomal *TC0101441* was elevated in patients with rAFS III and IV, compared to patients with rAFS I and II.	1
LINC01133 [24]	Tissue: 36 eutopic and 54 ectopic EM of mixed entities + 42 controls	Human: Tissue	Higher expressed in ectopic tissues compared to eutopic tissue of women with EM and to controls	
		**Animal studies**		
BANCR [25]		Rat: Tissue Serum	Inhibition of *BANCR* could significantly reduce the volume of the ectopic lesions	
H19 [26]	4 human ovarian lesions; 20 mice implanted with H19 infected ecESCs+20 controls	Mice: ecESCs from human ovarian lesions	Volume and mass of subcutaneous lesions were reduced in a H19 knockdown mouse model of EM.	

EM: endometriosis; CIN: cervical intraepithelial neoplasia; rAFS: revised American Fertility Society AUC: area under ROC curve; GCs: Granulosa Cells.

1, small sample size (n<30/group); 2, no comprehensive clinical information (i.e. rAFS stage, lesion entities, menstrual cycle phase); 3, EM-free controls are not appropriate (i.e. CIN patients, no laparoscopic proof); 4, Not all relevant tissues analyzed (i.e. eutopic tissues of EM-free controls, eutopic and ectopic tissues of EM patients)

References

1. Huang, H.; Zhu, Z.; Song, Y., Downregulation of lncrna uca1 as a diagnostic and prognostic biomarker for ovarian endometriosis. *Rev Assoc Med Bras (1992)* **2019,** 65, (3), 336-341.
2. Jiang, L.; Wan, Y.; Feng, Z.; Liu, D.; Ouyang, L.; Li, Y.; Liu, K., Long Noncoding RNA UCA1 Is Related to Autophagy and Apoptosis in Endometrial Stromal Cells. *Front Oncol* **2020,** 10, 618472.
3. Yu, J.; Chen, L. H.; Zhang, B.; Zheng, Q. M., The modulation of endometriosis by lncRNA MALAT1 via NF-kappaB/iNOS. *Eur Rev Med Pharmacol Sci* **2019,** 23, (10), 4073-4080.
4. Liu, H.; Zhang, Z.; Xiong, W.; Zhang, L.; Du, Y.; Liu, Y.; Xiong, X., Long non-coding RNA MALAT1 mediates hypoxia-induced pro-survival autophagy of endometrial stromal cells in endometriosis. *J Cell Mol Med* **2019,** 23, (1), 439-452.
5. Liang, Z.; Chen, Y.; Zhao, Y.; Xu, C.; Zhang, A.; Zhang, Q.; Wang, D.; He, J.; Hua, W.; Duan, P., miR-200c suppresses endometriosis by targeting MALAT1 in vitro and in vivo. *Stem Cell Res Ther* **2017,** 8, (1), 251.
6. Li, Y.; Liu, Y. D.; Chen, S. L.; Chen, X.; Ye, D. S.; Zhou, X. Y.; Zhe, J.; Zhang, J., Down-regulation of long non-coding RNA MALAT1 inhibits granulosa cell proliferation in endometriosis by up-regulating P21 via activation of the ERK/MAPK pathway. *Mol Hum Reprod* **2019,** 25, (1), 17-29.
7. Du, Y.; Zhang, Z.; Xiong, W.; Li, N.; Liu, H.; He, H.; Li, Q.; Liu, Y.; Zhang, L., Estradiol promotes EMT in endometriosis via MALAT1/miR200s sponge function. *Reproduction* **2019,** 157, (2), 179-188.
8. Wang, M.; Hao, C.; Huang, X.; Bao, H.; Qu, Q.; Liu, Z.; Dai, H.; He, S.; Yan, W., Aberrant Expression of lncRNA (HOXA11-AS1) and Homeobox A (HOXA9, HOXA10, HOXA11, and HOXA13) Genes in Infertile Women With Endometriosis. *Reprod Sci* **2018,** 25, (5), 654-661.
9. Ghazal, S.; McKinnon, B.; Zhou, J.; Mueller, M.; Men, Y.; Yang, L.; Mueller, M.; Flannery, C.; Huang, Y.; Taylor, H. S., H19 lncRNA alters stromal cell growth via IGF signaling in the endometrium of women with endometriosis. *EMBO Mol Med* **2015,** 7, (8), 996-1003.
10. Liu, S.; Xin, W.; Tang, X.; Qiu, J.; Zhang, Y.; Hua, K., LncRNA H19 Overexpression in Endometriosis and its Utility as a Novel Biomarker for Predicting Recurrence. *Reprod Sci* **2020,** 27, (9), 1687-1697.
11. Liu, Z.; Liu, L.; Zhong, Y.; Cai, M.; Gao, J.; Tan, C.; Han, X.; Guo, R.; Han, L., LncRNA H19 over-expression inhibited Th17 cell differentiation to relieve endometriosis through miR-342-3p/IER3 pathway. *Cell Biosci* **2019,** 9, 84.
12. Liu, Y.; Ma, J.; Cui, D.; Fei, X.; Lv, Y.; Lin, J., LncRNA MEG3-210 regulates endometrial stromal cells migration, invasion and apoptosis through p38 MAPK and PKA/SERCA2 signalling via interaction with Galectin-1 in endometriosis. *Mol Cell Endocrinol* **2020,** 513, 110870.
13. Lin, D.; Huang, Q.; Wu, R.; Dai, S.; Huang, Z.; Ren, L.; Huang, S.; Chen, Q., Long non-coding RNA AFAP1-AS1 promoting epithelial-mesenchymal transition of endometriosis is correlated with transcription factor ZEB1. *Am J Reprod Immunol* **2019,** 81, (1), e13074.
14. Liu, Y.; Huang, X.; Lu, D.; Feng, Y.; Xu, R.; Li, X.; Yin, C.; Xue, B.; Zhao, H.; Wang, S.; Ma, Y.; Jia, C., LncRNA SNHG4 promotes the increased growth of endometrial tissue outside the uterine cavity via regulating c-Met mediated by miR-148a-3p. *Mol Cell Endocrinol* **2020,** 514, 110887.
15. Cui, L.; Chen, S.; Wang, D.; Yang, Q., LINC01116 promotes proliferation and migration of endometrial stromal cells by targeting FOXP1 via sponging miR-9-5p in endometriosis. *J Cell Mol Med* **2021,** 25, (4), 2000-2012.
16. Holdsworth-Carson, S. J.; Churchill, M.; Donoghue, J. F.; Mortlock, S.; Fung, J. N.; Sloggett, C.; Chung, J.; Cann, L.; Teh, W. T.; Campbell, K. R.; Luwor, R.; Healey, M.; Montgomery, G.; Girling, J. E.; Rogers, P. A. W., Elucidating the role of long intergenic non-coding RNA 339 in human endometrium and endometriosis. *Mol Hum Reprod* **2021,** 27, (3).

17. Mai, H.; Xu, H.; Lin, H.; Wei, Y.; Yin, Y.; Huang, Y.; Huang, S.; Liao, Y., LINC01541 Functions as a ceRNA to Modulate the Wnt/beta-Catenin Pathway by Decoying miR-506-5p in Endometriosis. *Reprod Sci* **2021,** 28, (3), 665-674.
18. Wang, H.; Ni, C.; Xiao, W.; Wang, S., Role of lncRNA FTX in invasion, metastasis, and epithelial-mesenchymal transition of endometrial stromal cells caused by endometriosis by regulating the PI3K/Akt signaling pathway. *Ann Transl Med* **2020,** 8, (22), 1504.
19. Wang, H.; Sha, L.; Huang, L.; Yang, S.; Zhou, Q.; Luo, X.; Shi, B., LINC00261 functions as a competing endogenous RNA to regulate BCL2L11 expression by sponging miR-132-3p in endometriosis. *Am J Transl Res* **2019,** 11, (4), 2269-2279.
20. Wang, S.; Yi, M.; Zhang, X.; Zhang, T.; Jiang, L.; Cao, L.; Zhou, Y.; Fang, X., Effects of CDKN2B-AS1 on cellular proliferation, invasion and AKT3 expression are attenuated by miR-424-5p in a model of ovarian endometriosis. *Reprod Biomed Online* **2021,** 42, (6), 1057-1066.
21. Zhang, C.; Wu, W.; Ye, X.; Ma, R.; Luo, J.; Zhu, H.; Chang, X., Aberrant expression of CHL1 gene and long non-coding RNA CHL1-AS1, CHL1-AS2 in ovarian endometriosis. *Eur J Obstet Gynecol Reprod Biol* **2019,** 236, 177-182.
22. Qiu, J. J.; Lin, X. J.; Zheng, T. T.; Tang, X. Y.; Zhang, Y.; Hua, K. Q., The Exosomal Long Noncoding RNA aHIF is Upregulated in Serum From Patients With Endometriosis and Promotes Angiogenesis in Endometriosis. *Reprod Sci* **2019,** 26, (12), 1590-1602.
23. Qiu, J. J.; Lin, Y. Y.; Tang, X. Y.; Ding, Y.; Yi, X. F.; Hua, K. Q., Extracellular vesicle-mediated transfer of the lncRNA-TC0101441 promotes endometriosis migration/invasion. *Exp Cell Res* **2020,** 388, (1), 111815.
24. Yotova, I.; Hudson, Q. J.; Pauler, F. M.; Proestling, K.; Haslinger, I.; Kuessel, L.; Perricos, A.; Husslein, H.; Wenzl, R., LINC01133 Inhibits Invasion and Promotes Proliferation in an Endometriosis Epithelial Cell Line. *International Journal of Molecular Sciences* **2021,** 22, (16), 8385.
25. Zhu, M. B.; Chen, L. P.; Hu, M.; Shi, Z.; Liu, Y. N., Effects of lncRNA BANCR on endometriosis through ERK/MAPK pathway. *Eur Rev Med Pharmacol Sci* **2019,** 23, (16), 6806-6812.
26. Liu, S.; Xin, W.; Lu, Q.; Tang, X.; Wang, F.; Shao, W.; Zhang, Y.; Qiu, J.; Hua, K., Knockdown of lncRNA H19 suppresses endometriosis in vivo. *Braz J Med Biol Res* **2021,** 54, (4), e10117.
